# Distinct Patterns of Clinical Features and Cardiac Biomarker Elevation in Community-Acquired Pneumonia and COVID-19 Pneumonia

**DOI:** 10.3390/pathogens15070676

**Published:** 2026-06-26

**Authors:** Murimisi Mukansi, Helen C. Steel, Theresa M. Rossouw, Ismail Kalla, Colin Menezes, Martin Nieuwoudt, Ronald Anderson, Charles Feldman

**Affiliations:** 1 Division of Pulmonology and Critical Care, Helen Joseph Hospital, Johannesburg 2006, South Africa; 2 Department of Internal Medicine, Faculty of Health Sciences, University of the Witwatersrand, Johannesburg 2006, South Africa; 3 Department of Immunology, School of Clinical Medicine, Faculty of Health Sciences, University of Pretoria, Pretoria 0001, South Africa; 4 Institute for Biomedical Engineering, Faculty of Engineering, University of Stellenbosch, Stellenbosch 7599, South Africa

**Keywords:** cardiac biomarkers, cardiovascular events, community-acquired pneumonia, comorbidities, COVID-19 pneumonia, HIV

## Abstract

No previous sub-Saharan studies have compared patients with community-acquired pneumonia (CAP) and COVID-19 pneumonia, the focus of this study. Consecutive adult patients hospitalized with CAP (*n* = 59) or COVID-19 pneumonia (*n* = 74) were compared regarding multiple characteristics, including cardiac biomarkers. In multivariable logistic regression analysis, differences were noted among various clinical features. Troponin I concentrations (*p* = 0.00028) and the Troponin I/NT-pro BNP ratio (*p* = 0.00048) were significantly higher in COVID-19 compared with CAP. After adjustment for age, these differences remained significant (troponin I *p* = 0.0019; ratio *p* = 0.00054), while BNP concentrations were now higher in CAP (*p* = 0.009). PCA demonstrated that BNP and NT-pro BNP contributed most strongly to the dominant cardiac biomarker signature, suggesting shared cardiopulmonary stress across both diseases. Exploratory subgroup analyses suggested higher troponin I levels among people living with HIV and COVID-19, although interaction modelling did not demonstrate significant effect modification by HIV status. Both CAP and COVID-19 pneumonia were associated with evidence of cardiac stress; however, COVID-19 demonstrated a relatively stronger myocardial injury signature characterized by higher troponin I concentrations and an increased Troponin I/NT-pro BNP ratio while CAP had evidence of greater hemodynamic cardiac strain, as evidenced by the higher levels of BNP. The findings suggest that the mechanisms of cardiac involvement may differ between viral and bacterial respiratory infections.

## 1. Introduction

Acute respiratory infections are increasingly recognized to induce systemic inflammatory responses that may contribute to cardiac injury. Understanding whether distinct pathogens produce different patterns of cardiac stress may provide insight into infection-specific mechanisms of organ injury. In this regard, both community-acquired pneumonia (CAP) and COVID-19 pneumonia are multisystem diseases with the respiratory tract as the port of entry [1,2,3], in which cardiovascular events (CVEs) have been noted to occur. CVEs represent one of the major extrapulmonary manifestations of pneumonia, which contributes to mortality and morbidity [1,2,3]. They develop in up to a quarter of cases, contribute considerably to both short- and long-term cardiac dysfunction, and may occur at the time of diagnosis of pneumonia or during convalescence [1,2,3]. CVEs can occur in patients with or without underlying cardiovascular disease (CVD) and/or its risk factors [4,5]. Notably, CVD itself represents a risk factor for both CAP and COVID-19, forming a bidirectional relationship [1,6,7,8,9,10].

The pathophysiology of the CVEs involves both pathogen-mediated and/or host-mediated myocardial damage [1,11]. Host-mediated events are related to the systemic inflammatory/immune response to these infections by mechanisms that have not been fully elucidated [3,12]. In approximately 20–30% of COVID-19 cases, myocardial injury is indicated by elevated levels of N-terminal pro-B-type natriuretic peptide (NT-Pro BNP) and cardiac troponin I (cTnI) isoenzymes, which are indicative of major CVEs [13,14].

SARS-CoV-2 infection continues to present major clinical, diagnostic, and therapeutic challenges, with some 20% of patients with SARS-CoV-2 infection developing severe complications, including COVID-19 pneumonia [15,16,17]. Notably, the clinical and radiological features of patients with COVID-19 pneumonia are very similar to those of patients with CAP, irrespective of bacterial or viral etiology, which also frequently occurred during the pandemic [18]. Clearly, there is a need to differentiate between the two types of infection, because they require different forms of management and therapy [19]. However, while the treatment of CAP is clearly outlined in most guidelines, that of COVID-19 pneumonia is still under development [20,21].

Cardiovascular complications were noted to occur in patients with COVID-19 very early on in the pandemic [1,4,12,18]. Early detection, treatment, and prevention of cardiovascular events will likely contribute to an improved outcome not only in CAP patients but also in COVID-19 pneumonia [22,23]. Although the COVID-19 pandemic is over, SARS-CoV-2 pathogens and their frequently emerging variants still circulate in most countries, continuing to cause COVID-19 infections [24,25] and are predicted to be present globally for the foreseeable future. Therefore, CVE will remain a problem for patients with these infections, worthy of ongoing investigation [24,25,26].

Some studies have compared clinical and laboratory parameters and CVEs in patients with CAP and those with COVID-19 pneumonia; however, there are no data from sub-Saharan Africa among patients with a high prevalence of human immunodeficiency virus (HIV) infection [19,27,28,29,30,31]. Accordingly, the purpose of the current study was to investigate and compare demographic, clinical, and routine laboratory characteristics, as well as systemic inflammatory and cardiovascular biomarker profiles in patients with CAP or COVID-19 pneumonia.

## 2. Patients and Methods

### 2.1. Study Population

This was a prospective study conducted at two centers, namely, the Helen Joseph Hospital (HJH; University of the Witwatersrand, Johannesburg, RSA), which enrolled CAP patients in the pre-COVID-19 era, and the Steve Biko Academic Hospital (SBAH; University of Pretoria, Pretoria, RSA), which recruited patients with COVID-19 pneumonia. The study enrolled CAP cases between August 2019 and March 2020 and COVID-19 cases between March and June 2021. Cases enrolled (*n* = 133) were consecutive adult patients ≥ 18 years old, hospitalized with either CAP (*n* = 59) or COVID-19 pneumonia (*n* = 74). A control group (*n* = 15) comprising healthy HIV-uninfected individuals was included in the study. This was considered to be the minimum number required to establish baseline laboratory values in this population group.

The study components were approved by the appropriate Human Research Ethics Committees (University of the Witwatersrand: reference M180382, 29 May 2018; University of Pretoria: reference 247/2020, 30 July 2020). Written informed consent was obtained from all participants before the commencement of the study. The diagnosis of pneumonia was based on the clinical signs and symptoms (cough, sputum expectoration, pleuritic chest pain, dyspnea, and/or fever) and a radiologically confirmed pulmonary infiltrate. Immunosuppressed individuals were included. The exclusion criteria were as follows: (1) nosocomial infection, occurring more than 48 h after admission and/or hospitalization in the preceding 90 days; (2) children below 18 years of age; (3) aspiration pneumonitis; and (4) a diagnosis of tuberculosis (TB).

SARS-CoV-2 infection was confirmed using a polymerase chain reaction (PCR) test on nasopharyngeal swabs, in accordance with the recommendations of the National Institute for Communicable Diseases, Johannesburg, RSA [32]. The CAP patients presented before the pandemic, prior to the availability of tests for SARS-CoV-2; therefore, no testing to rule out COVID-19 was conducted or indicated in that group. The severity of CAP was based on the CURB-65 score (a 5-point severity of illness score) [33], while that of COVID-19 pneumonia was based on the WHO severity score, these being the most common severity of illness scores used for the individual infections. The latter score subdivided COVID-19 cases into mild, moderate, and severe or critical [34]. An inflammation score was calculated based on the work of Zhu et al. [35].

A detailed medical history and examination were obtained on admission for all enrolled patients, including the current symptoms and duration, as well as previous illnesses and data on relevant chronic medications. HIV status, as well as CD4+ T-cell count and viral load, were determined on admission, mainly based on case notes together with patient history. Additional patients were confirmed to be HIV-positive by PCR.

### 2.2. Laboratory Testing

Sampling for all the investigations was performed on admission of the patients to the hospital. Routine laboratory testing was performed as part of the standard of care, including a full blood count, urea and electrolytes, C-reactive protein (CRP), and procalcitonin (PCT). Samples were processed at the National Health Laboratory Services in Johannesburg or Pretoria, Gauteng, RSA. In addition, levels of study-specific biomarkers B-natriuretic peptide (BNP), creatine kinase-MB (CK-MB), endocan-1, NT-pro BNP, and troponin I were measured using a Milliplex^®^ MAP Human Cardiovascular Disease panel (Merck, KgaA, Darmstadt, Germany). The biomarkers were analyzed using a Bio-Plex suspension array platform (Bio-Rad, Hercules, CA, USA). All methods were conducted according to the manufacturers’ guidelines. Analysis of all these assays was conducted at the Department of Immunology, University of Pretoria. For the measurement of cardiac and inflammatory biomarkers, plasma was processed and stored at −80 °C prior to analysis. The rationale for choosing these cardiac biomarkers is that they are the ones most commonly studied and have variously been shown to be useful markers of cardiac stress and/or cardiac injury, including myocardial damage.

### 2.3. Statistical Analyses

Statistical analyses were performed using Stata 17 (Stata Statistical Software: Release 17. StataCorp LLC, College Station, TX, USA). Data were expressed as the mean ± standard deviation (SD) for parametric parameters and the median ± interquartile range (IQR) for the parameters with a non-Gaussian distribution. Categorical/binary values are presented as counts and percentages, with comparisons performed using Pearson’s chi-square or Fisher’s exact tests. Comparisons of continuous variables were made using the parametric tests (paired and unpaired Student’s t-test) and non-parametric tests (Kruskal–Wallis test with the post hoc Dunn test for unpaired samples and the Wilcoxon signed-rank test for paired samples), with *p*-values ≤ 0.05 considered statistically significant. Adjustment for multiple comparisons was not performed in this analysis, because univariate analyses were used for variable screening rather than hypothesis testing. Spearman’s correlation with Bonferroni correction for multiple comparisons was performed for clinical and laboratory variables of interest.

A multivariable logistic regression model was constructed to identify variables independently associated with COVID-19 pneumonia versus CAP. To address missingness and reduce instability from overlapping predictors, we built a parsimonious model using clinically relevant variables and variables associated with group status in univariate analysis (*p* < 0.10) and avoided collinearity and excessive missing data. Derived and highly overlapping variables were not entered together. Assumptions of logistic regression were evaluated prior to model fitting. Independence of observations and binary outcome requirements were satisfied. Multicollinearity was assessed using variance inflation factors, which were all <2, indicating no significant collinearity between predictors. Model convergence occurred without evidence of separation. Continuous predictors were assessed for plausibility of linearity in the logit.

To address potential confounding by comorbidities, additional parsimonious linear regression models were performed, adjusting for age, sex, HIV status [36,37], and individual cardiovascular comorbidities (hypertension, diabetes mellitus, chronic heart disease, and renal dysfunction) to assess the robustness of the cardiac biomarker associations.

To explore the multivariable structure of cardiac biomarker responses, principal component analysis (PCA) was performed using standardized biomarker values (z-score transformation). Principal components were extracted using the eigenvalue decomposition of the covariance matrix. The first two principal components (PC1 and PC2) were retained for visualization and interpretation based on the proportion of variance explained. PCA results were visualized using biplots displaying both individual patient scores and biomarker loading vectors. This allowed identification of biomarker clusters and assessment of separation between CAP, COVID-19 pneumonia and control groups.

## 3. Results

Table 1 shows the demographic information, underlying comorbid conditions, clinical findings, and antihypertensive/antidiabetic therapy of the patients with CAP or with COVID-19 pneumonia at initial presentation. Patients with CAP were significantly younger than patients with COVID-19. Among the comorbidities, the frequency of HIV positivity was significantly higher in patients with CAP compared with those with COVID-19 pneumonia (45.8% versus 32.4%; *p* = 0.032). In contrast, the frequencies of neoplastic diseases, chronic heart disease, diabetes mellitus, chronic obstructive airway disease, hypertension, and coronary artery disease were significantly higher in the COVID-19 group. Concomitant with the higher rate of comorbidities, the use of antihypertensive medication (single agent, dual therapy, or triple therapy) and antidiabetic medication was significantly more common in patients with COVID-19 versus CAP (28/74 [37.9%] versus 2/59 [3.4%]; *p* < 0.0001). In addition, one patient in each group was on aspirin and/or a statin.

Patients with COVID-19 pneumonia had a longer duration of respiratory symptoms at presentation and were more likely to have dyspnea than patients with CAP (Table 1). In contrast, cough and fever were significantly more common in the CAP group. In patients admitted with CAP, systolic and diastolic blood pressure, as well as the respiratory rate, were lower, while the heart rate was higher. Patients with CAP and COVID-19 generally had mild or moderate disease, and while the severity scores cannot directly be compared, only one patient with CAP, and seven with COVID-19 pneumonia (*p* = 0.065), had severe disease.

There were several significant differences in the routine laboratory parameters between the two groups, with important differences in a lower peripheral oxygen saturation (SpO_2_) but higher creatinine, white cell count, CRP and PCT in the CAP group, compared with the COVID-19 group (Table 2). The results show that patients with CAP and HIV (with an HIV viral load [VL] available) had significantly higher VLs than patients with COVID-19 pneumonia and HIV. Significantly more patients admitted with CAP had a detectable VL, i.e., >50 copies/mL (CAP 13/16 vs. COVID-19 10/24; *p* = 0.014), and numerically more had levels above 1000 copies/mL (CAP 10/16 vs. COVID-19 7/24; *p* = 0.053) and 100,000 copies/mL (CAP 4/16 vs. COVID 1/24; *p* = 0.073), although the latter two differences did not reach statistical significance.

**Figure 1:**
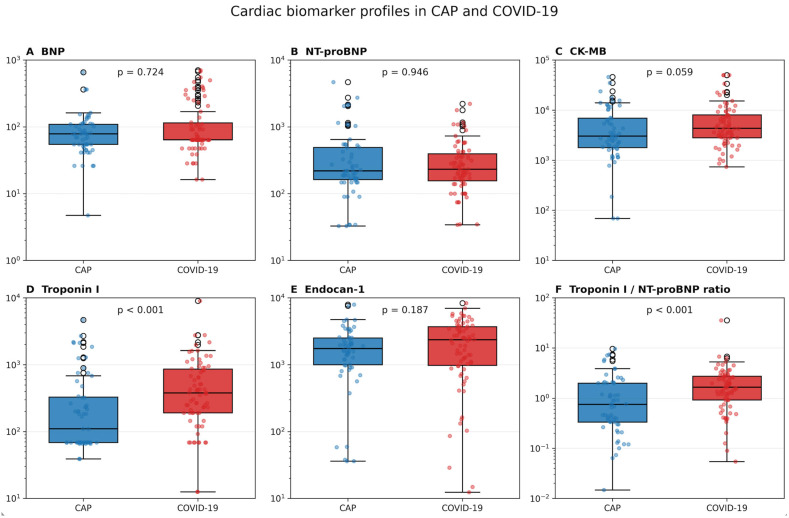
Cardiac biomarker profiles in CAP and COVID-19. Boxplots showing distributions of circulating cardiac biomarkers in patients with community-acquired pneumonia (CAP) and COVID-19 pneumonia. Panels show (**A**) BNP, (**B**) NT-pro BNP, (**C**) CK-MB, (**D**) troponin I, (**E**) endocan-1, and (**F**) Troponin/NT-pro BNP ratio. Blue and red filled circles represent individual participants with CAP and COVID-19, respectively, while open black circles indicate outliers. NT-pro BNP and troponin I are displayed on a logarithmic scale due to right-skewed distributions. *p*-values were calculated using the Mann–Whitney U test. Abbreviations: BNP: brain natriuretic peptide; CK-MB: creatine kinase–myoglobin binding; NT-pro BNP: N-terminal pro-brain natriuretic peptide.

**Figure 2:**
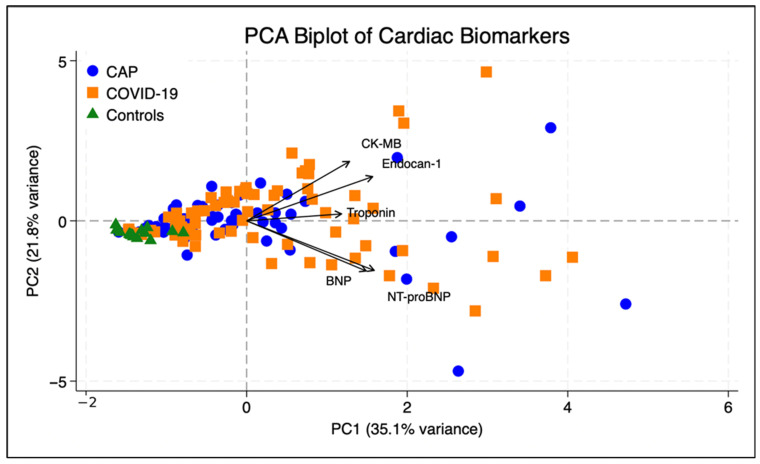
Principal component analysis biplot of cardiac biomarkers in CAP, COVID-19 pneumonia and controls. Principal component analysis was performed on standardized BNP, NT-pro BNP, CK-MB, troponin I and endocan-1 values. Points represent individual patients and are colored by diagnostic group. Arrows represent biomarker loading vectors, illustrating the contribution of each variable to the two principal components. The first principal component (PC1) reflects a myocardial injury axis driven predominantly by troponin I and CK-MB, whereas the second principal component (PC2) is influenced primarily by NT-pro BNP, BNP and endocan-1, consistent with cardiac strain and endothelial activation.

Supplementary Table S1 shows the univariate comparison of the cardiac biomarkers in patients with CAP, COVID-19 pneumonia, and healthy control individuals. NT-pro BNP, CK-MB, and endocan-1 were significantly higher in patients with CAP compared with the control group. The results were similar for those with COVID-19 pneumonia, except that troponin I was also higher than in the control group. Significantly higher troponin I levels were observed in individuals with COVID-19 pneumonia compared with CAP (*p* = 0.00028). NT-pro BNP, BNP, CK-MB and endocan-1 concentrations did not differ significantly between groups. The Troponin I/NT-pro BNP ratio was also significantly higher in COVID-19 (*p* = 0.00048), suggesting relatively greater myocardial injury in COVID-19 compared with CAP. Figure 1 shows the distributions of circulating cardiac biomarkers in patients with CAP and COVID-19 pneumonia. As indicated below, in PCA, BNP and NT-pro BNP contributed most strongly to the dominant cardiac biomarker signature, indicating that overall cardiac strain was present in both conditions (Figure 2).

In multivariable logistic regression analysis, shorter duration of symptoms, higher white blood cell count, and absence of dyspnea were independently associated with CAP compared with COVID-19 (Table 3). Conversely, longer duration of symptoms, presence of dyspnea, and a history of hypertension were more strongly associated with COVID-19. The final model demonstrated good discrimination between the two groups (AUC 0.90).

We explored whether HIV modified the relationship between key discriminating variables and pneumonia group by adding HIV interaction terms one at a time to the logistic regression model. No significant interaction terms were identified (Supplementary Table S2), and these were therefore not retained in the final model.

To explore the multivariable structure of cardiac biomarker responses, PCA was performed using standardized values of BNP, NT-pro BNP, CK-MB, troponin I and endocan-1 (Figure 2). The first two principal components accounted for a substantial proportion of the overall variance in the dataset. The first principal component (PC1) was primarily driven by troponin I and CK-MB, consistent with a myocardial injury signature, whereas the second principal component (PC2) was more strongly influenced by NT-pro BNP, BNP and endocan-1, reflecting cardiac strain and endothelial activation. Visualization of PCA scores demonstrated partial separation between CAP, COVID-19 pneumonia, and control participants, indicating distinct biomarker patterns associated with the different clinical groups. Individuals with COVID-19 tended to cluster toward higher values along the myocardial injury axis, whereas individuals with CAP showed a distribution more consistent with cardiac strain. Controls clustered closer to the origin of the PCA space, reflecting lower biomarker levels. Together, these findings suggest that cardiac injury in COVID-19 pneumonia and CAP may involve overlapping but partially distinct pathophysiological mechanisms, with myocardial injury and cardiac strain contributing differently across disease groups.

### 3.1. Subgroup Analysis by Age

Because cardiac biomarkers are influenced by age, age-adjusted linear regression models were performed (Table 4). After adjustment for age, troponin I remained significantly higher in COVID-19 compared with CAP (*p* = 0.0019). In contrast, BNP was significantly higher in CAP after age adjustment (*p* = 0.0090). The Troponin I/NT-pro BNP ratio remained significantly higher in COVID-19 (*p* = 0.00054), suggesting a relatively greater myocardial injury signature in COVID-19 pneumonia compared with CAP. NT-pro BNP and CK-MB did not differ significantly between the groups after adjustment for age. Sex was not independently associated with disease group and inclusion of this variable did not materially alter the associations observed for age or cardiac biomarkers.

### 3.2. Subgroup Analysis by Sex

Subgroup analysis by sex within the combined CAP and COVID-19 pneumonia groups revealed significantly higher levels of troponin I in women (Table 5). In the individual groups, no significant differences were seen between the two sexes with CAP, while in the COVID-19 group, troponin I was significantly higher in women (*p* = 0.011) (Supplementary Table S3).

### 3.3. Subgroup Analysis by HIV Status

A subgroup analysis was performed to explore whether HIV infection influenced cardiac biomarker profiles within each disease group. Among patients with CAP, no significant differences in cardiac biomarkers were observed between individuals with and without HIV infections. In contrast, among patients with COVID-19 pneumonia, individuals with HIV demonstrated significantly higher levels of BNP (*p* = 0.009), troponin I (*p* = 0.016), and endocan-1 (*p* = 0.019) and non-significantly higher levels of NT-pro BNP (*p* = 0.077) than patients with COVID-19 and without HIV (Supplementary Table S4). No significant associations were detected between the cardiac markers of people with HIV infection and either CD4+ T-cell count or VL. These findings should be interpreted cautiously given the exploratory nature of the analysis.

An interaction model was constructed to evaluate whether HIV infection modified the association between disease group and troponin I levels. Although HIV-positive patients with COVID-19 pneumonia demonstrated higher troponin I levels in subgroup analyses, the interaction between HIV status and disease group was not statistically significant (*p* = 0.10), suggesting limited evidence for effect modification, likely reflecting limited power (Supplementary Table S5).

### 3.4. Subgroup Analysis by Comorbidities

Additional models adjusting individually for major cardiovascular comorbidities yielded similar results, with troponin I and the Troponin I/NT-pro BNP ratio remaining significantly higher in COVID-19 pneumonia compared with CAP across these models (Supplementary Table S6).

In a multivariable linear regression model including disease group, sex, age, and HIV status, COVID-19 remained independently associated with higher troponin I levels compared with CAP (fold-change 1.94, 95% CI 1.21–3.11, *p* = 0.0065), whereas sex, age, and HIV status were not independently associated with troponin I (Supplementary Table S7).

The inflammatory score (Supplementary Data Section C) correlated well with the WHO COVID-19 severity score in the categories mild, moderate, and severe, but, unfortunately, due to the small number of CAP patients with all variables recorded, further analysis was not conducted.

The correlations between the cardiac biomarkers and various other clinical and laboratory parameters are shown in the Supplementary Data Section D and Table S8.

Although elevations of cardiac biomarkers were noted, none of the patients were documented to have had any clinically apparent CVEs. In addition, only one patient in the CAP group died, with no deaths in the COVID-19 pneumonia group. Therefore, the association of the biomarkers with the occurrence of clinical CVEs or mortality could not be fully evaluated.

## 4. Discussion

These findings suggest that pathogen-specific host responses may influence the pattern of cardiac injury observed during acute respiratory infections. Several features distinguished patients with CAP from those with COVID-19 pneumonia, including their demographic profile, comorbidities, signs and symptoms, routine laboratory parameters, and cardiac biomarkers. These included a younger CAP cohort, more comorbidities in the COVID-19 cohort, aside from HIV, which was more common in the CAP cases, a longer duration of respiratory symptoms prior to hospital presentation and dyspnea more common in patients with COVID-19. In the CAP group, there was a lower peripheral oxygen saturation, higher WBC count, CRP, and PCT, as well as a lower creatinine clearance. There were raised cardiac biomarkers in both pneumonia groups compared with the control cohort, while troponin I was higher in the COVID-19 cases than in the CAP cases. Since the SARS-CoV-2 virus is predicted to continue circulating and causing infection globally for the foreseeable future, with the intermittent emergence of variants, studies describing the occurrence of CVEs in association with these infections remain relevant.

### 4.1. Age

Patients with CAP were younger and were more commonly HIV-infected than the patients with COVID-19 pneumonia, who were older and had more general comorbidities. This differs from the findings in an Italian study, which indicated non-COVID-19 patients as being relatively older with significant comorbidities [38]. Furthermore, COVID-19 has been observed to be highly prevalent, particularly severe, and potentially lethal in the elderly population [39]. These differences may be due to an association between the socioeconomic development levels of different countries, HIV prevalence, and the population age gap, with age also related to the severity of the illness and mortality [39,40,41,42].

### 4.2. Comorbidities

The literature confirms the finding that a global increase in morbidity and mortality occurs in patients with COVID-19 with comorbidities, compared with those without comorbidities [43,44,45]. Hypertension, diabetes mellitus, and chronic heart disease are among the most prevalent comorbidities, similar to those indicated in Table 1 of the current study [46]. The increase in the risk of SARS-CoV-2 infection doubles in cases of multimorbidity [46]. In CAP, similarly, there is an associated increase in the incidence of the disease and worse outcomes [47]. The VL was significantly elevated in the CAP group compared with the COVID-19 group of patients (Table 2). Previous studies have documented an increased risk of CAP in patients with poorly controlled HIV, while the relationship documented in patients with HIV and COVID-19 includes both the severity of COVID-19 and the mortality [48,49].

### 4.3. Symptoms

The duration of symptoms in this cohort was significantly longer in COVID-19 pneumonia patients compared with the CAP patients, confirming the findings of Zhao et al. [50]. However, cough and fever were more frequent in the CAP group, as opposed to dyspnea, which was more significant in the COVID-19 cohort. In another study, the symptoms were similar between the two groups, with fever and cough common in both CAP and COVID-19 pneumonia, which is in contrast with Chen et al., who observed that fever and cough were more common in their patients with COVID-19 than in those with CAP [51,52].

### 4.4. Laboratory Parameters

On admission, the arterial blood gas analysis showed significantly different abnormalities between the COVID-19 group and the CAP group. The need for oxygen was more evident in the CAP group compared with the COVID-19 group. There was significant respiratory alkalosis, with compensation, as evidenced by elevated pH and lower bicarbonate in the COVID-19 group. These observations have been corroborated in many studies where the blood acid–base status ranges from respiratory to metabolic acidosis or alkalosis, with respiratory alkalosis in the majority [53,54,55]. Various pathophysiological mechanisms are involved, including fever, dysregulation of the renin–angiotensin system, multi-organ inflammation, thrombogenesis, respiratory tract infections, and carotid body suppression, with the predominance of each contributing to the underlying acid–base shift at different stages of COVID-19 [54,55,56,57,58].

The CAP group had significantly higher leukocyte counts, as well as levels of CRP and PCT, possibly underscoring the differences between bacterial and viral infections. The trend of the first three parameters is in keeping with the observations described in the literature, with the elevation of these inflammatory markers pointing towards a higher severity of infection [59,60,61]. However, in the study by Zhao et al., there was no significant difference in these biomarkers between the SARS-CoV-2-seronegative and -seropositive patients with pneumonia [51].

Furthermore, lactate was significantly higher in patients with CAP compared with the COVID-19 group, as corroborated by several authors [62,63]. Unlike in CAP, lactate remains near-normal in patients with COVID-19 despite an elevated lactate dehydrogenase. Mitochondrial function is likely preserved in SARS-CoV-2 infection, despite a hyperinflammatory state, unlike in non-COVID-19 CAP [62,64].

The creatinine clearance was lower in the patients with CAP compared with those with COVID-19 pneumonia. Renal dysfunction is a sign of pneumonia progression in both patients with CAP and those with COVID-19, as indicated in several studies [65,66,67,68,69,70,71,72,73,74,75,76,77,78], which describe high blood urea nitrogen and serum creatinine levels as markers of renal dysfunction. Furthermore, there is a glomerular filtration rate reduction, usually measured by laboratory creatinine clearance. In addition, in severe COVID-19, the elevation of serum creatinine is associated with an increased number of leukocytes, low levels of lymphocytes and platelets, as well as coagulopathy [77,79,80,81].

### 4.5. Cardiac Biomarkers

The cardiac biomarkers were mostly elevated in both patients with CAP and COVID-19 relative to the control group (Supplementary Table S1), as had been suggested previously [82,83,84]. However, in this study, cardiac biomarker profiles differed between patients with COVID-19 pneumonia and CAP. Although both conditions demonstrated evidence of cardiac stress, COVID-19 was characterized by significantly higher troponin I concentrations and a higher Troponin I/NT-pro BNP ratio compared with CAP, even after adjustment for age. In contrast, NT-pro BNP levels were similar between the two groups. PCA further demonstrated that natriuretic peptides contributed most strongly to the dominant cardiac biomarker signature across the cohort, suggesting that cardiopulmonary stress represents a common feature of both diseases. However, the relative predominance of troponin I elevation in COVID-19 indicates a stronger myocardial injury signal compared with CAP. These findings are consistent with accumulating evidence that COVID-19 may be associated with myocardial injury mediated by microvascular inflammation, endothelial dysfunction, and immune-mediated mechanisms, whereas cardiac involvement in CAP is more frequently related to systemic inflammatory stress and sepsis-associated cardiomyopathy.

Importantly, elevated cardiac biomarker levels may be associated with non-cardiac reasons, as well as with cardiac stress and/or cardiac injury, including the occurrence of myocardial infarction. So, although no clinically apparent CVEs were noted among this cohort of patients, it does not preclude the occurrence of cardiac damage.

Exploratory analyses examining the potential influence of HIV infection suggested that PLWH with COVID-19 may demonstrate higher troponin I and BNP levels compared with HIV-negative patients. However, interaction modelling did not demonstrate a statistically significant interaction between HIV status and disease group, suggesting that these findings should be interpreted cautiously given the limited sample size. Nevertheless, these observations raise the possibility that chronic immune activation and endothelial dysfunction associated with HIV infection may exacerbate myocardial stress during acute viral infection, a hypothesis that warrants further investigation in larger cohorts.

### 4.6. Cardiac Biomarker Correlations

Regarding the various correlations, there were significant positive correlations between some of the cardiac biomarkers, in particular, BNP and NT-pro BNP with each other and with troponin I. There were also positive correlations between the following variables: age with heart rate, SpO_2_ with both heart rate and serum sodium, pH with HCO_3_^−^, and white blood cell count with creatinine (range of rho values: 0.61–0.70; corresponding range of *p*-values: 0.049–0.018). In contrast, the following variables showed significant negative correlations: age with SpO_2_ and troponin I; diastolic blood pressure with lactate; heart rate with SpO_2_, CK-MB, and troponin I; pH with serum potassium; platelet lymphocyte ratio with BNP, NT-pro BNP, and troponin I; CRP with troponin I; PCT with serum sodium; and ALT with CKMB (range of rho values: −0.62–0.83; corresponding range of *p*-values: 0.041–0.003) (Supplementary Table S8, correlation matrix).

### 4.7. Strengths and Potential Limitations

As far as we are aware, this is the first study in sub-Saharan Africa to compare the characteristics of patients with CAP and those with COVID-19 pneumonia and to describe the systemic inflammatory and cardiac biomarker differences between the two groups, both in the presence and absence of concurrent HIV infection. The findings of the current study will add to the few global studies, which have compared clinical and laboratory parameters between these two groups of patients [22,27,31,38,59,85,86,87,88].

However, this study also has some potential limitations. First, it was conducted in two different institutions, which may be associated with some bias and may also not be generalizable to the rest of South Africa or sub-Saharan Africa. However, both institutions are teaching hospitals, situated approximately 50 km apart, with HJH affiliated with the University of the Witwatersrand, in Johannesburg, and SBAH affiliated with the University of Pretoria, in Pretoria, and both fall under the direction of the Gauteng Department of Health (GDOH). As such, the admission criteria and various aspects of patient admission, investigation and management at the two institutions would be very similar. The study was a collaborative investigation between long-term collaborating research groups at each of the two centers. Routine investigations were performed at the National Health Laboratory Services of the GDH and the specialized investigations at the University of Pretoria Immunology Department by the same individual, and, as such, there would be identical laboratory methods, platforms, calibration standards and sample processing procedures utilized.

Second, the two cohorts were not recruited simultaneously, but at different time intervals, which may have added significant bias. However, this was unavoidable due to measures instituted to control the COVID-19 pandemic, including initial halting of routine research by institutional ethics committees (such as, for example, the recruitment of CAP cases) and the subsequent overwhelming of hospital wards with COVID-19 cases during the pandemic, with the admission of very few CAP cases, both during the pandemic and for a considerable period thereafter. Third, while systemic inflammatory and cardiac biomarkers were measured in both groups, there was no systematic evaluation for the presence of clinically apparent CVEs, although it appears that none of the primary physicians were alerted to the risk of their development or appeared to be concerned about such events.

Fourth, data were not collected on antiretroviral therapy in the CAP group; therefore, we were not able to determine the possible impact of antiretroviral therapy on any of the findings, particularly the changes in cardiac biomarkers. Fifth, there was a similar lack of severe infections in both groups and only one death in the CAP group; therefore, a comparison of characteristics in the context of severe versus mild-to-moderate disease was not possible, nor was it possible to determine factors associated with mortality. Sixth, there was no long-term follow-up of the patients to allow for an evaluation of the occurrence of longer-term CVEs and associated changes in the levels of cardiovascular biomarkers. Seventh, a small group of 15 healthy volunteers, without HIV, was recruited as a control group, this being considered the minimum number of controls needed to set the baseline laboratory values in this population group. Recruiting larger numbers was precluded by costs. Eight, in this study, no systematic investigation was undertaken to identify microbial causes in the CAP group, or for the presence of co-infecting or superinfecting microorganisms in either of the study groups, which may have impacted the study findings. Lastly, since only in-hospital patients were recruited, the results cannot be extrapolated to outpatients.

## 5. Conclusions

Taken together, these findings suggest that while both CAP and COVID-19 pneumonia may be associated with measurable cardiac stress, the relative pattern of biomarker elevation differs between the two conditions. COVID-19 appears to manifest a relatively greater myocardial injury signature, whereas CAP may reflect a pattern more consistent with cardiopulmonary strain associated with systemic infection. These differences may reflect distinct underlying pathophysiological mechanisms and highlight the potential value of biomarker profiling in distinguishing patterns of cardiac involvement in infectious diseases.

## Figures and Tables

**Table 1:** Demographics, comorbidities, clinical characteristics, and therapy among patients with CAP versus COVID-19 pneumonia.

	CAP (*n* = 59)	COVID-19 Pneumonia (*n* = 74)	*p*-Value
Demographic Information			
Age (years) (mean ± SD)	44.3 (±13.3)	53.1 (±13.1)	<0.001
Sex (*n*)	27 males: 32 females	36 males: 38 females	0.811
Comorbidities [*n* (%)]			
HIV	27 (45.8)	24 (32.4)	0.032
Neoplastic disease	0 (0)	3 (4.1)	0.021
Chronic kidney disease	1 (1.7)	4 (5.4)	0.065
Chronic heart disease	1 (1.7)	8 (10.8)	0.001
Diabetes mellitus	5 (8.5)	20 (27.0)	<0.001
COPD	1 (1.7)	5 (6.8)	0.005
Hypertension	4 (6.8)	27 (36.5)	<0.001
Symptoms			
Number of days symptomatic	5 (3–6)	7 (5–9)	0.001
Cough	56/56 (100%)	43/54 (79.6%)	<0.001
Fever	27/56 (48.2%)	21/54 (38.9%)	<0.001
Dyspnea	29/56 (51.8%)	45/54 (83.3%)	<0.001
Sputum expectoration	36/56 (64.3%)	Not recorded	
Clinical features			
Systolic BP (mmHg)	112.5 (98.5–131)	126 (117–139)	0.003
Diastolic BP (mmHg)	67.5 (55.5–82.5)	79 (69–86)	0.002
Respiratory rate (breaths/min)	22 (19–24)	26 (22–30)	0.002
Heart rate (beats/min)	112 (103–127)	100 (90–109.5)	<0.001
Medication			
Antihypertensive agents	2/59 (3.4%)	13/74 (17.6%)	
Antidiabetic agents	0/59 (0%)	15/74 (20.3%)	
			<0.0001

The results are reported as the median (interquartile range) unless indicated otherwise. Abbreviations: BP: blood pressure; CAP: community-acquired pneumonia; COPD: chronic obstructive pulmonary disease; COVID-19: COVID-19 pneumonia; HIV: human immunodeficiency virus.

**Table 2:** Routine laboratory parameters of patients with CAP versus COVID-19 pneumonia.

Laboratory Parameters	CAP (*n* = 59)	COVID-19 Pneumonia (*n* = 74)	*p*-Value
Arterial blood gas			
pH	7.43 (7.38–7.47)	7.47 (7.42–7.51)	0.004
SpO_2_	78.25 (66.05–94.5)	93 (90–96)	<0.001
HCO_3_	21.15 (20–23.8)	20 (18–22.5)	0.027
Lactate	2 (1.3–2.9)	1.35 (0.9–1.7)	0.004
PaO_2_/FiO_2_	199 (149–280)	183.2 (104.4–391.7)	0.236
HIV			
CD4+ T-cell count (cells/mm^3^)	197 (156–298.5)	212 (117–296)	0.381
HIV viral load (copies/mL)	1850 (69.5–81,500)	20 (20–12,738)	0.006
Hematology			
WBC	13.48 (9.20–18.99)	8.54 (6.85–10.86)	<0.001
PMN	11.46 (6.23–17.02)	7.32 (5.29–9.55)	0.040
Lymphocytes	1.44 (1.04–1.93)	1.11 (0.75–1.50)	0.072
PMN/lymphocyte ratio	6.78 (4.07–11.9)	6.84 (4.27–10.74)	0.490
Platelets	262.5 (192.5–313.5)	261.5 (184–352)	0.362
Platelet/lymphocyte ratio	151 (80–238)	277 (162–382)	0.010
Inflammatory biomarkers			
CRP	235.5 (109–310)	113 (69.5–215.5)	0.004
PCT	0.81 (0.68–62.03)	0.09 (0.04–0.40)	0.003
Urea and electrolytes			
Na^+^ (mean ± SD)	134 ± 5	136 ± 3.8	0.027
K^+^	4.0 (3.5–4.5)	4.4 (4.0–5.1)	<0.001
BUN	5.6 (3.8–9.7)	5.3 (3.7–8.0)	0.263
Creatinine	86 (64–124)	71 (59–95)	0.032
Creatinine clearance * (mL/min)	60 (42–60)	60 (60–60)	<0.001
HbA1c	6.1 (5.7–7.2)	6.3 (5.8–7.7)	0.333
Liver function			
AST	46 (27–104)	39 (24–61)	0.126
ALT	45 (27–72)	28 (18–47)	0.011
ALP	83 (64–119)	81 (68–116)	0.381
GGT	53.5 (28–99)	71 (42–133)	0.069

* The maximum creatinine clearance was taken as 60 mL/min. The results are reported as the median (interquartile range) unless indicated otherwise. Abbreviations: ALP: alkaline phosphatase; ALT: alanine transaminase; AST: aspartate transaminase; BUN: blood urea nitrogen; CRP: C-reactive protein; CD4: cluster of differentiation 4; FiO_2_: fraction of inspired oxygen; GGT: gamma-glutaryl transaminase; HCO_3_: bicarbonate; HIV: human immunodeficiency virus; K^+^: potassium; Na^+^: sodium; PaO_2_: partial pressure of oxygen; PCT: procalcitonin; PMN: polymorphonuclear cells; SpO_2_: peripheral oxygen saturation; WBC: white blood cells.

**Table 3:** Multivariable logistic regression identifying variables associated with CAP versus COVID-19 pneumonia.

Variable	Odds Ratio	95% CI	*p*-Value
Age	0.95	0.88–1.01	0.100
Days symptomatic	0.76	0.61–0.94	0.013
WBC	1.27	1.07–1.50	0.005
Dyspnea	0.20	0.05–0.86	0.030
Hypertension	0.17	0.03–0.98	0.048

Abbreviations: CI = confidence interval; WBC = white blood count; % = percent. Model performance: *n* = 74 (COVID-19 *n* = 36; CAP *n* = 38), Pseudo R^2^ = 0.43, Area under ROC curve = 0.90.

**Table 4:** Age-adjusted analysis of cardiac biomarkers between patients with CAP and COVID-19 pneumonia.

Biomarker	CAP Median (IQR)	COVID Median (IQR)	Univariate *p*-Value	Age-Adjusted *p*-Value
Troponin I	109.9 (68.6–328.6)	377.4 (190.4–865.7)	0.00028	0.0019
NT-pro BNP	219.5 (163.1–492.9)	232.1 (155.4–395.9)	0.946	0.744
BNP	78.3 (54.7–109.1)	63.9 (63.9–114.7)	0.724	0.0090
CK-MB	3023.6 (1790.1–6916.2)	4316.2 (2814.1–8066.1)	0.0586	0.320
Troponin/NT-pro BNP ratio	0.75 (0.34–2.00)	1.66 (0.93–2.75)	0.00048	0.00054

The results are reported as pg/mL. Abbreviations: BNP: brain natriuretic peptide; CK-MB: creatine kinase-myoglobin binding; NT-pro BNP: N-terminal pro-brain natriuretic peptide.

**Table 5:** Analysis of cardiac biomarkers by sex in CAP and COVID-19 pneumonia cases combined.

Biomarkers	Male (*n* = 63)	Female (*n* = 69)	*p*-Value
BNP	63.89 (54.74–91.76)	81.00 (54.74–118.62)	0.110
NT-pro BNP	203.85 (139.11–424.22)	253.03 (168.17–424.90)	0.350
CK-MB	3414.84 (1973.84–7148.17)	3791.43 (2322.82–9295.14)	0.340
Troponin I	190.35 (68.59–514.39)	321.68 (119.94–879.03)	0.048
Endocan-1	1588.82 (731.94–3136.50)	2054.73 (1295.80–3460.84)	0.068

The results are reported as pg/mL. Abbreviations: BNP: brain natriuretic peptide; CK-MB: creatine kinase-myoglobin binding; NT-pro BNP: N-terminal pro-brain natriuretic peptide.

## Data Availability

Data will be made available upon reasonable request.

## Supplementary Materials

The following supporting information can be downloaded at https://www.mdpi.com/article/10.3390/pathogens15070676/s1, Table S1: Univariate comparison of cardiovascular biomarkers in participants with CAP, COVID-19, and in healthy control individuals; Table S2: Analysis of levels of cardiac biomarkers in CAP and COVID-19 groups by HIV status; Table S3: Analysis of levels of cardiac biomarkers in CAP and COVID-19 pneumonia groups by sex; Table S4: Exploratory linear regression models for cardiac biomarkers comparing patients with and without HIV within the COVID-19 group; Table S5: HIV interaction terms tested one at a time; Table S6: Additional parsimonious linear regression models for cardiac biomarkers comparing COVID-19 vs. CAP, adjusting for age and comorbidities; Table S7: Additional parsimonious linear regression models for troponin I comparing COVID-19 versus CAP, adjusting for age, sex, and human immunodeficiency virus (HIV); Table S8: Correlation matrix.
