# Supplementary material for: Distinct Patterns of Clinical Features and Cardiac Biomarker Elevation in Community-Acquired Pneumonia and COVID-19 Pneumonia
Source: Pathogens. 2026 Jun 26;15(7):676. doi: 10.3390/pathogens15070676 (PMC13414867; doi:10.3390/pathogens15070676)
Supplement: Supplementary file 1 [file pathogens-15-00676-s001.zip › pathogens-4153945-supplementary.pdf]

Supplementary material:

Distinct Patterns of Clinical Features and Cardiac Biomarker Elevation in Community-Acquired Pneumonia and COVID-19 Pneumonia

Murimisi Mukansi [1,2,*], Helen C. Steel [3], Theresa M. Rossouw [3], Ismail Kalla [2], Colin Menezes [2], Martin Nieuwoudt [4], Ronald Anderson [3] and Charles Feldman [2]

[1] Division of Pulmonology and Critical Care, Helen Joseph Hospital, Johannesburg 2006, South Africa
[2] Department of Internal Medicine, Faculty of Health Sciences, University of the Witwatersrand, Johannesburg 2006, South Africa
[3] Department of Immunology, School of Clinical Medicine, Faculty of Health Sciences, University of Pretoria, Pretoria 0001, South Africa
[4] Institute for Biomedical Engineering, Faculty of Engineering, University of Stellenbosch, Stellenbosch 7599, South Africa
* Correspondence: murimisi.mukansi@wits.ac.za

A) Univariate analysis of cardiac biomarkers

Supplementary Table S1. Univariate comparison of cardiovascular biomarkers in participants with CAP, COVID-19, and in healthy control individuals.

Biomarkers	CAP (n = 59)	COVID-19 (n = 74)	Controls (n = 15)	CAP vs. COVID	CAP vs. Controls	COVID vs. Controls
BNP	78.26 (54.74–109.10)	63.89 (63.89–116.21)	75.41 (57.87–91.60)	0.377	0.4702	0.388
NT-pro BNP	219.47 (157.95–496.29)	232.08 (155.39–401.96)	128.56 (34.29–186.89)	0.466	**<0.001**	**<0.001**
CK-MB	3023.57 (1741.37–7037.52)	4316.19 (2805.97–8115.60)	2239.70 (712.44–4215.44)	**0.028**	**0.038**	**0.001**
Troponin-I	109.94 (68.59–335.6)	377.38 (190.35–879.03)	129.77 (88.75–232.97)	**<0.001**	0.310	**0.001**
Endocan-1	1735.86 (988.60–2522.52)	2354.55 (963.00–3716.34)	368.45 (19.45–812.69)	0.123	**<0.001**	**<0.001**
Troponin I/NT-pro BNP ratio	0.75 (0.34–2.00)	1.66 (0.93-2.75)	1.24 (0.96-2.00)	**<0.001**	0.062	0.402

The results are reported as pg/mL. Values in bold denote significance. Note that p-values are different from Figure 1 since the figure is on a log scale. Abbreviations: BNP: brain natriuretic peptide; CKMB: creatine kinase–myoglobin binding; NT-pro BNP: N-terminal pro-brain natriuretic peptide.

B) Subgroup analysis

Supplementary Table S2. Analysis of levels of cardiac biomarkers in CAP and COVID-19 groups by HIV status.

Biomarker	CAP			COVID-19 Pneumonia		
	**HIV-Positive (n = 27)**	**HIV-Negative (n = 23)**	**p-Value**	**HIV-Positive (n = 24)**	**HIV-Negative (n = 49)**	**p-Value**
BNP	78.26 (41.39–109.10)	78.26 (54.74–109.10)	0.244	104.23 (63.89–297.11)	63.89 (56.06–91.76)	**0.009**
NT-pro BNP	203.85 (146.99-544.40)	226.87 (168.17–547.46)	0.196	272.56 (184.10–440.63)	209.31 (139.11–363.38)	0.077
CK-MB	2769.82 (1741.37–6521.26)	3086.83 (1838.89–7037.52)	0.469	3946.62 (2983.94–7674.85)	3697 (2177.50–4832.24)	0.130
Troponin I	109.94 (68.59–683.16)	183.96 (68.59–572.26)	0.282	458.64 (292.39–1215.38)	268.68 (145.13–654.37)	**0.016**
Endocan-1	1735.86 (910.67–2566.36)	2657.02 (979.06–2076.91)	0.389	2657.02 (1472.44–3842.20)	1924.86 (637.42–3440.47)	**0.019**

Values in bold denote significance. Abbreviations: BNP: B-type natriuretic peptide; CAP: community-acquired pneumonia; CK-MB: creatinine kinase–myoglobin binding; COVID-19: coronavirus disease-19; n: number; NT-pro BNP: N-terminal prohormone of brain natriuretic peptide.

Supplementary Table S3. Analysis of levels of cardiac biomarkers in CAP and COVID-19 pneumonia groups by sex.

Biomarkers	CAP			COVID-19 Pneumonia		
	**Male (n = 27)**	**Female (n = 31)**	**p-Value**	**Male (n = 36)**	**Female (n = 38)**	**p-Value**
BNP	69.81 (44.87–109.10)	81.00 (54.74–111.51)	0.178	63.89 (63.89–91.76)	71.30 (63.89–232.92)	0.075
NT-pro BNP	203.85 (168.17–547.46)	226.87 (146.99–496.29)	0.429	232.08 (134.71–359.28)	262.80 (184.10–424.90)	0.155
CK-MB	2418.95 (1149.60-5390.09)	3276.22 (2065.44–11,640.56)	0.054	3946.62 (2983.94–7674.85)	4681.20 (2708.19–9295.14)	0.451
Troponin I	68.59 (68.59–477.80)	183.96 (68.59–335.60)	0.256	240.62 (119.94–553.52)	458.64 (250.19–951.45)	**0.005**
Endocan-1	1588.82 (910.67–2119.70)	1901.95 (1295.73–3114.76)	0.191	1554.34 (661.78–3374.17)	2490.41 (1295.80–3844.90)	0.065

Values in bold denote significance. Abbreviations: BNP: B-type natriuretic peptide; CAP: community-acquired pneumonia; CK-MB: creatinine kinase–myoglobin binding; COVID-19: coronavirus disease-19; n: number; NT-pro BNP: N-terminal prohormone of brain natriuretic peptide.

Supplementary Table S4. Exploratory linear regression models for cardiac biomarkers comparing patients with and without HIV within the COVID-19 group.

Biomarker	Model	n	Adjusted fold-change (HIV+ vs HIV-)	95% CI	p-value
Troponin I	Age adjusted	73	1.96	1.09 to 3.51	**0.025**
Troponin I	Age + hypertension	65	2.00	1.09 to 3.67	**0.025**
Troponin I	Age + diabetes mellitus	65	2.10	1.14 to 3.89	**0.019**
Troponin I	Age + chronic heart disease	65	2.05	1.10 to 3.80	**0.024**
Troponin I	Age + chronic kidney disease	65	1.98	1.06 to 3.70	**0.032**
NT-proBNP	Age adjusted	73	1.40	0.90 to 2.19	0.134
NT-proBNP	Age + hypertension	65	1.31	0.82 to 2.10	0.247
NT-proBNP	Age + diabetes mellitus	65	1.35	0.84 to 2.16	0.209
NT-proBNP	Age + chronic heart disease	65	1.34	0.87 to 2.07	0.178
NT-proBNP	Age + chronic kidney disease	65	1.38	0.86 to 2.20	0.176
BNP	Age adjusted	73	1.61	1.04 to 2.49	**0.034**
BNP	Age + hypertension	65	1.52	0.97 to 2.39	0.065
BNP	Age + diabetes mellitus	65	1.52	0.97 to 2.38	0.066
BNP	Age + chronic heart disease	65	1.53	0.99 to 2.38	0.058
BNP	Age + chronic kidney disease	65	1.56	1.00 to 2.44	0.050
CK-MB	Age adjusted	73	1.41	0.89 to 2.25	0.145
CK-MB	Age + hypertension	65	1.27	0.81 to 2.01	0.292
CK-MB	Age + diabetes mellitus	65	1.29	0.81 to 2.05	0.284
CK-MB	Age + chronic heart disease	65	1.30	0.84 to 2.02	0.241
CK-MB	Age + chronic kidney disease	65	1.32	0.83 to 2.09	0.241
Troponin I / NT-proBNP ratio	Age adjusted	73	1.39	0.82 to 2.36	0.213
Troponin I / NT-proBNP ratio	Age + hypertension	65	1.53	0.89 to 2.61	0.122
Troponin I / NT-proBNP ratio	Age + diabetes mellitus	65	1.56	0.91 to 2.68	0.107

Troponin I / NT-proBNP ratio	Age + chronic heart disease	65	1.52	0.90 to 2.59	0.117
Troponin I / NT-proBNP ratio	Age + chronic kidney disease	65	1.44	0.86 to 2.41	0.166

Outcome variables were log-transformed before modelling.

Positive fold-change values indicate higher biomarker concentrations in HIV-positive than HIV-negative patients with COVID-19.

Models include HIV status, age, and one additional comorbidity at a time.

These analyses are exploratory because of the small sample size.

Abbreviations: BNP: brain natriuretic peptide; CKMB: creatine kinase–myoglobin binding; HIV: human immunodeficiency virus; NT-pro BNP: N-terminal pro-brain natriuretic peptide.

Values in bold indicate significance

Supplementary Table S5: HIV interaction terms tested one at a time.

**Interaction term**	**P-value**
HIV × Age	0.852
HIV × Days symptomatic	0.156
HIV × WBC	0.387
HIV × Dyspnea	0.495
HIV × Hypertension	0.827

Abbreviations: HIV; human immunodeficiency virus; WBC: white blood count

There is no evidence of any significant HIV interaction for the main predictors.

Supplementary Table S6. Additional parsimonious linear regression models for cardiac biomarkers comparing COVID-19 vs CAP, adjusting for age and comorbidities.

**Biomarker**	**Model**	**n**	**Adjusted fold-change (COVID vs CAP)**	**95% CI**	**p-value**
Troponin I	Age adjusted	127	2.05	1.31 to 3.21	**0.002**
Troponin I	Age + hypertension	119	1.73	1.07 to 2.81	**0.027**
Troponin I	Age + diabetes mellitus	119	1.92	1.20 to 3.07	**0.007**
Troponin I	Age + chronic heart disease	119	1.99	1.24 to 3.20	**0.005**
Troponin I	Age + chronic kidney disease	119	2.05	1.29 to 3.27	**0.003**
NT-proBNP	Age adjusted	127	0.94	0.66 to 1.35	0.744
NT-proBNP	Age + hypertension	119	0.86	0.58 to 1.28	0.464
NT-proBNP	Age + diabetes mellitus	119	0.93	0.64 to 1.36	0.703
NT-proBNP	Age + chronic heart disease	119	0.85	0.60 to 1.22	0.380
NT-proBNP	Age + chronic kidney disease	119	0.97	0.67 to 1.40	0.856
BNP	Age adjusted	127	1.47	1.10 to 1.95	**0.009**

BNP	Age + hypertension	119	1.50	1.11 to 2.03	**0.008**
BNP	Age + diabetes mellitus	119	1.51	1.13 to 2.02	**0.006**
BNP	Age + chronic heart disease	119	1.45	1.09 to 1.93	**0.012**
BNP	Age + chronic kidney disease	119	1.52	1.14 to 2.01	**0.004**
CK-MB	Age adjusted	127	1.21	0.83 to 1.78	0.320
CK-MB	Age + hypertension	119	1.16	0.78 to 1.75	0.461
CK-MB	Age + diabetes mellitus	119	1.33	0.90 to 1.97	0.156
CK-MB	Age + chronic heart disease	119	1.22	0.82 to 1.81	0.318
CK-MB	Age + chronic kidney disease	119	1.29	0.87 to 1.90	0.203
Troponin I / NT-proBNP ratio	Age adjusted	127	2.18	1.41 to 3.36	**<0.001**
Troponin I / NT-proBNP ratio	Age + hypertension	119	2.00	1.25 to 3.21	**0.004**
Troponin I / NT-proBNP ratio	Age + diabetes mellitus	119	2.06	1.31 to 3.25	**0.002**
Troponin I / NT-proBNP ratio	Age + chronic heart disease	119	2.33	1.50 to 3.63	**<0.001**
Troponin I / NT-proBNP ratio	Age + chronic kidney disease	119	2.13	1.39 to 3.26	**<0.001**

Outcome variables were log-transformed before modelling.
Positive fold-change values indicate higher biomarker concentrations in COVID-19 than in CAP.

Models include disease group (COVID-19 vs CAP), age, and one additional comorbidity at a time.

Comorbidities examined: hypertension, diabetes mellitus, chronic heart disease, and chronic kidney disease.
Abbreviations: BNP: brain natriuretic peptide; CKMB: creatine kinase–myoglobin binding; NT-pro BNP: N-terminal pro-brain natriuretic peptide.
Values in bold denote significance.

Supplementary Table S7: Additional parsimonious linear regression models for Troponin I comparing COVID-19 versus CAP, adjusting for age, gender, and human immunodeficiency virus (HIV)

**Variable**	**Fold-change**	**95% CI**	**p-value**
COVID-19	1.94	1.21 to 3.11	0.0065
Gender (female)	1.47	0.95 to 2.28	0.0849
Age	1.00	0.98 to 1.02	0.883
HIV	1.28	0.80 to 2.07	0.304

Values in bold denote significance.
After adjusting for gender, age, and HIV:

- COVID-19 remained significantly associated with higher Troponin I

- about 1.94-fold higher than CAP
- Gender was no longer statistically significant
  - There is only a trend toward higher troponin in females
- Age was not significant
- HIV was not significant

C) Inflammation score

Methods. An inflammation score was calculated based on the work of Zhu et al. [35]. Briefly, the score comprises four serum and cellular circulating inflammatory markers, namely, the CRP, WBC, platelet count, and granulocyte-to-lymphocyte ratio. Each value is calculated based on 10 tiles of each biomarker level. The highest (7–10) and lowest (1–4) deciles are scored as positive and negative from 1 to 4, respectively, and deciles 5–6 are scored as zero. The total score is the sum of the four biomarkers and ranges from -16 to 16.

Results. The CAP group (n=10) had a mean score of 0 (SD ±6.36) with the lowest score of -7 and the highest score of 10. The COVID-19 group (n=44) had a mean score of -0.39 (SD ±6.16) with the lowest score of -16 and the highest score of 11. The inflammatory score correlated well with the WHO COVID-19 severity score: mild disease had a mean of -6.25 (SD ±8.66); moderate disease had a mean of -0.56 (SD ±5.58), and severe disease had a mean of 2.5 (SD 5.84). Due to the small number of CAP patients with all variables recorded, further analysis was not conducted.

D) Correlations

Regarding the various correlations, there were significant positive correlations between some of the cardiac biomarkers, in particular, BNP and NT-pro BNP with each other and with troponin I. There were also positive correlations between the following variables: age with heart rate, SpO_2 with both heart rate and serum sodium, pH with HCO_3^-, and white blood cell count with creatinine (range of rho values: 0.61-0.70; corresponding range of p-values: 0.049-0.018). In contrast, the following variables showed significant negative correlations: age with SpO_2 and troponin I; diastolic blood pressure with lactate; heart rate with SpO_2, CK-MB, and troponin I; pH with serum potassium; platelet lymphocyte ratio with BNP, NT-pro BNP, and troponin I; CRP with troponin I; PCT with serum sodium; and ALT with CKMB (range of rho values: -0.62-0.83; corresponding range of p-values: 0.041-0.003) (Supplementary Table 8, correlation matrix).

Supplementary Table S8. Correlation matrix.

	Age	Systolic BP	Diastolic BP	Respiratory Rate	Heart Rate	pH	SpO_2	HCO_3	Lactate	WBC	PMN	PLR	CRP	PCT	Na	K	Creatinine	ALT	BNP	ProBNP	CK-MB	TroponinI	Endocan-1
Age	1.000																						
Systolic BP	0.053	1.000																					
Diastolic BP	0.090	**0.872**	1.000																				
Respiratory Rate	0.092	−0.041	−0.174	1.000																			
Heart Rate	**0.608**	0.076	0.014	0.190	1.000																		
pH	−0.106	0.181	−0.041	0.384	0.153	1.000																	
SpO_2	**−0.627**	−0.152	−0.117	0.055	**−0.835**	0.266	1.000																
HCO_3	−0.051	0.232	0.113	0.303	−0.241	**0.706**	0.507	1.000															
Lactate	−0.151	−0.291	**−0.654**	0.104	0.085	0.383	0.039	0.176	1.000														
WBC	0.073	−0.032	−0.183	0.205	0.365	0.119	−0.046	0.060	0.216	1.000													
PMN	0.320	0.023	−0.187	0.305	0.529	0.196	−0.211	0.110	0.313	**0.946**	1.000												
PLR	0.339	−0.237	−0.055	0.219	0.201	0.087	−0.018	−0.395	−0.395	−0.227	−0.109	1.000											
CRP	0.429	0.558	0.423	0.317	0.427	0.151	−0.282	0.005	−0.023	0.424	0.565	0.223	1.000										
PCT	0.408	0.561	0.398	−0.128	0.413	−0.132	−0.584	−0.301	−0.437	0.305	0.419	0.150	0.637	1.000									
Na	−0.369	−0.439	−0.412	0.482	−0.555	0.114	**0.624**	0.452	0.228	−0.178	−0.214	−0.014	−0.404	**−0.717**	1.000								
K	0.214	−0.112	0.163	−0.307	0.039	**−0.774**	0.139	−0.533	**−1.067**	0.361	0.233	−0.027	0.149	0.233	−0.298	1.000							
Creatinine	0.051	0.378	0.277	−0.193	0.276	−0.014	−0.129	0.055	0.055	**0.688**	**0.679**	−0.211	0.524	0.533	−0.336	0.318	1.000						
ALT	0.041	0.324	0.347	0.460	0.547	0.410	−0.270	0.147	−0.235	0.136	0.182	0.246	0.223	0.187	−0.100	−0.237	0.174	1.000					
BNP	−0.408	0.098	0.070	−0.504	−0.466	0.226	0.401	0.450	0.047	−0.158	−0.302	**−0.647**	−0.536	−0.392	0.040	−0.222	−0.122	−0.335	1.000				
ProBNP	−0.517	0.082	−0.037	−0.269	−0.560	0.087	0.375	0.439	0.267	−0.218	−0.327	**−0.809**	−0.529	−0.506	0.360	−0.315	−0.119	−0.373	**0.833**	1.000			
CK-MB	−0.174	0.174	0.242	−0.214	**−0.757**	−0.264	0.494	0.188	−0.129	−0.346	−0.400	−0.218	0.027	−0.246	0.287	0.137	−0.147	**−0.691**	0.321	0.446	1.000		
Troponin I	**−0.686**	−0.183	−0.238	−0.354	**−0.637**	−0.037	0.445	0.181	0.196	−0.278	−0.460	**−0.661**	**−0.792**	−0.548	0.361	−0.243	−0.299	−0.392	**0.800**	**0.888**	0.287	1.000	
Endocan-1	−0.256	0.361	0.311	−0.178	−0.519	−0.342	0.183	0.137	0.055	−0.355	−0.346	−0.373	−0.073	0.000	0.328	−0.059	0.184	−0.236	0.135	0.518	0.564	0.346	1.000

Abbreviations: ALT: alanine transaminase; BNP: brain-type natriuretic peptide; CK-MB: creatinine kinase–myoglobin binding; CRP: C-reactive protein; HCO_3: bicarbonate; K: potassium; Na: sodium; NTproBNP: N-terminal prohormone of brain natriuretic peptide; PCT: procalcitonin; PMN: polymorphonuclear leukocyte count; PLR: platelet lymphocyte ratio; SpO_2: peripheral oxygen saturation; WBC: white blood cell count. Annotations: Red indicates negative, and blue indicates positive correlations, with the shades indicating the strength of the correlation. The numbers in bold indicate significance.
